# The Hydrophilic Loop of *Arabidopsis* PIN1 Auxin Efflux Carrier Harbors Hallmarks of an Intrinsically Disordered Protein

**DOI:** 10.3390/ijms23116352

**Published:** 2022-06-06

**Authors:** Veronika Bilanovičová, Nikola Rýdza, Lilla Koczka, Martin Hess, Elena Feraru, Jiří Friml, Tomasz Nodzyński

**Affiliations:** 1 Mendel Centre for Plant Genomics and Proteomics, Central European Institute of Technology (CEITEC), Masaryk University, Kamenice 5, CZ-625 00 Brno, Czech Republic; 422960@mail.muni.cz (V.B.); nikolarydza@gmail.com (N.R.); koczka.lilla90@gmail.com (L.K.); martinnhess@email.cz (M.H.); 2 National Centre for Biomolecular Research, Faculty of Science, Masaryk University, Kamenice 5, CZ-625 00 Brno, Czech Republic; 3 Department of Plant Biotechnology and Bioinformatics, Ghent University, Technologiepark 71, 9052 Ghent, Belgium; elena.feraru@boku.ac.at (E.F.); jiri.friml@ist.ac.at (J.F.); 4 VIB-UGent Center for Plant Systems, Technologiepark 71, 9052 Ghent, Belgium; 5 Department of Applied Genetics and Cell Biology (DAGZ), Institute of Molecular Plant Biology (IMPB), University of Natural Resources and Life Sciences (BOKU), Muthgasse 18, 1190 Vienna, Austria; 6 Institute of Science and Technology (IST), 3400 Klosterneuburg, Austria

**Keywords:** PIN1, hydrophilic hoop, dimerization, intrinsic disorder, subcellular trafficking

## Abstract

Much of plant development depends on cell-to-cell redistribution of the plant hormone auxin, which is facilitated by the plasma membrane (PM) localized PIN FORMED (PIN) proteins. Auxin export activity, developmental roles, subcellular trafficking, and polarity of PINs have been well studied, but their structure remains elusive besides a rough outline that they contain two groups of 5 alpha-helices connected by a large hydrophilic loop (HL). Here, we focus on the PIN1 HL as we could produce it in sufficient quantities for biochemical investigations to provide insights into its secondary structure. Circular dichroism (CD) studies revealed its nature as an intrinsically disordered protein (IDP), manifested by the increase of structure content upon thermal melting. Consistent with IDPs serving as interaction platforms, PIN1 loops homodimerize. PIN1 HL cytoplasmic overexpression in *Arabidopsis* disrupts early endocytic trafficking of PIN1 and PIN2 and causes defects in the cotyledon vasculature formation. In summary, we demonstrate that PIN1 HL has an intrinsically disordered nature, which must be considered to gain further structural insights. Some secondary structures may form transiently during pairing with known and yet-to-be-discovered interactors.

## 1. Introduction

Plants have evolved a complex developmental mechanism using multiple signaling molecules. One of them, auxin, plays an essential role in the growth, division, differentiation of plant cells [1], and during the ontogenesis of the entire plant [2,3]. The critical elements in auxin cell-to-cell redistribution are plasma membrane-localized PIN FORMED (PIN) proteins [4,5]. Their polar localization in cells of various tissues [6] enables the directional efflux of auxin [7] resulting in local maxima that drive plant development [8,9,10].

Recent topology studies revealed a cytoplasmic orientation of the central hydrophilic loop (HL) connected by two groups of five helical transmembrane domains (TMDs) in PIN1–4 [11]. It is worth noting that when comparing amino acid sequences of multiple plant PINs, their HLs are more diverse than the relatively conserved TMD regions [12]. However, the HL is not devoid of discernible areas, and the literature about PIN sequence features reveals four highly conserved (HC) motifs, HC1–HC4, in that central loop [13]. This raises the question if the loop folds into a structure, maybe at least partially in regions harboring the conserved motifs separated by presumably less folded flexible linkers.

As already mentioned, the directionality of auxin transport correlates with the polar localization of PINs [6]. The asymmetric localization of those efflux carriers on the membranes of some cells is facilitated by vesicular trafficking in which clathrin-mediated endocytosis [14,15] machinery plays a role, as does the HL of PM PINs [13]. The large cytosolic loop of PIN1 was shown to interact with µ-adaptins, primarily through motif-containing Phe—165, which is essential for PIN1 endocytosis [16].

Additionally, PIN1 HL was also shown to contain phosphorylation [17,18] and ubiquitylation [19,20] motifs. Notably, phosphorylation and dephosphorylation of serine/threonine residues within the HL regulate polar delivery of the PIN protein as well as its efflux activity [21,22,23,24]. PIN1 phosphorylation results in enhanced apicalization of the protein in root vasculature cells of *Arabidopsis* seedlings. In contrast, detaching the phosphate groups facilitates a more rootward polarity of PIN1. The importance of HL phosphorylation and dephosphorylation was demonstrated on various knockout and overexpression lines of PINOID (PID) kinase as well as protein phosphatase 2A (PP2A) [7,21,25,26]. In addition, the PID mediated phosphorylation of the HL impacts PIN1 polarity and activity while the phosphate attachment by the D6 PROTEIN KINASE (D6PK) is necessary for PIN1 efflux activity [23,26]. Regarding PIN1 polarity regulation according to recent studies in *Arabidopsis*, peptidyl-prolyl *cis*/*trans* isomerase Pin1At catalyzes the *cis*/*trans* isomerization of the phosphorylated Ser/Thr residues preceding proline (pSer/Thr-Pro). The effect on PIN1 polarity of Pin1At overexpression is similar to PID kinase overexpression resulting in more apical and generally less polar PIN1 [27]. Notably, the links between the phosphate group attachment and the changes in PIN trafficking have not been mechanistically understood. For many proteins, it has been shown that phosphorylation can trigger alterations in the secondary structure of the protein [28]. Also, phosphorylation of Ser/Thr-Pro motifs and proline *cis* or *trans* isomerization constitute a major regulatory mechanism controlling ubiquitin-mediated proteolysis [29]. Overall, the results mentioned above link the HL structural changes in the vicinity of phosphorylation sites with the polar delivery of the whole PIN moiety. This also suggests the existence of folded regions in the HL that undergo unique rearrangements, for example, upon binding. It is conceivable that the HL harbors structural elements that interact with coat proteins during vesicle formation. It is also possible that the HL interacts with yet undiscovered protein players while the PIN is at the PM, forming complexes that promote structure formation in the regions of HL that are unstructured without those neighbor-interactions.

Another aspect that interconnects structure and polarity maintenance is limiting PIN PM diffusion away from the polar domain; this is presumably achieved through clustering of the individual efflux carriers [30,31]. Cysteine residues (C39 and C560) located in the linking loops between TMDs were involved in PIN2 distribution in the plasma membrane microdomains [32]. Notably, a recent study revealed the possible contribution of cysteines in forming PIN dimers [33]. Nevertheless, the partial participation of the hydrophilic loop was not dismissed. Dimerization of auxin efflux carriers could be a mechanism to slow their diffusion in the membrane, as would be their interaction with other protein partners that are more massive or are affixed by interaction with the cell wall. Indeed, digesting away the cell wall abolished PIN polar localization [34]. It is plausible that the HL could participate in PIN dimerization or generally serve as interaction platforms for other proteins, as shown for the above-mentioned µ-adaptins. 

Thus far, published information concerning the long PINs and the PIN1, as a prominent representative, delineate a somewhat modular approach for investigating the efflux carrier’s structure-function connections. Some studies are focused on the TMDs, while others explore motifs or simply phosphorylation sites in the HL. Although structural studies on the entire PIN would be ideal, obtaining the whole protein in sufficient amounts and purity for X-ray crystallography or Nuclear Magnetic Resonance (NMR) remains a challenge. Therefore, we decided to continue exploiting the modular approach of investigation. We focused on the HL fragment, as part of PIN1, that could be sufficiently expressed and purified for studies using physical chemistry and structural methods. Moreover, we also overexpressed PIN1 HL in the cytoplasm, investigating its effects on sub-cellular trafficking and plant development related to HL functionality.

## 2. Results

### 2.1. PIN1 HL Contains Unstructured and Structured Regions Resistant to Thermal Melting

**Figure 1:**
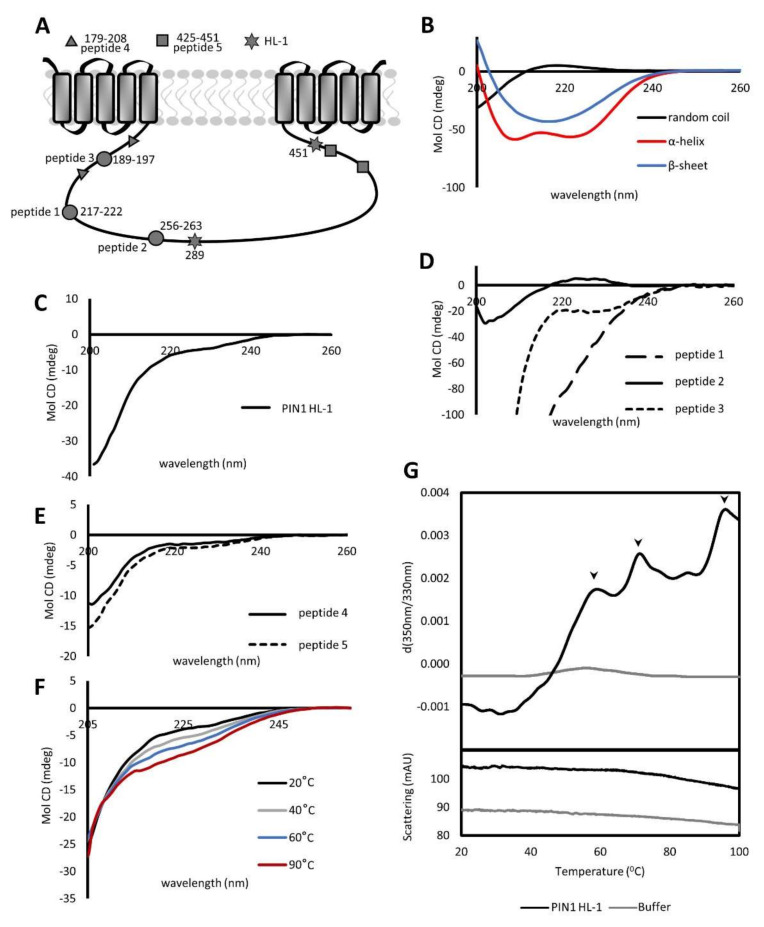
PIN1 hydrophilic loop contains thermally stable structured and unstructured parts reminiscent of an intrinsically disordered protein region. (**A**) Schematic representation of *A. thaliana* PIN1 protein topology. Two transmembrane domain groups are divided by a long hydrophilic loop. Grey circles indicate approximate positions of short peptides selected based on computer modeling for circular dichroism. Triangles and squares mark the beginning and the end of long peptides. Stars mark the start and the end amino acid of the hydrophilic loop used for most of the later biophysical analysis. (**B**) Circular dichroism spectra of poly–L–lysine at pH 7 and 11. At pH 7 poly–L–Lys shows spectra specific for unstructured protein (black line), compared with its spectra at pH 11 where it has exclusively helical (red line) or β-sheet composition after heating to 60 °C (blue line). (**C**–**E**) Circular dichroism spectra of PIN1 HL−1, short and long peptides. (**F**) Thermal melting CD spectra of PIN1 HL–1 were obtained at different temperatures ranging from 20 to 90 °C. The final spectrum is a representation of the average of three biological repetitions. (**G**) PIN1 HL–1 thermogram generated by nanoDSF indicating the presence of amino acid chain secondary structure in the Tyrosine surroundings (black line) overlaid with the buffer thermogram where no structure is detected (grey line). The *X*–axis presents the temperature and the *Y*–axis represents the first derivative of fluorescence intensity ratio 350 nm/330 nm. The bottom panel represents scattering data that do not detect protein aggregates.

It has been shown that *Arabidopsis* long PINs are polarly localized membrane proteins [35] with ten transmembrane domains separated in the middle by a long hydrophilic loop facing the cytoplasm (Figure 1A) [11]. Up to now, the protein has been poorly characterized structurally [13] because obtaining the whole PIN protein, and generally, PM proteins, for biochemical or structural studies, remains a challenge. However, since the long PINs contain a large cytoplasmic region, it is tempting to focus on this domain, express it separately from the full transporter, and treat it as a globular protein that is usually much easier to purify and characterize. 

A similar approach has worked in the case of the proline *cis-trans* isomerization in the vicinity of serine or threonine phosphorylation sites present in the PIN1 HL [27]. This study was conducted using ten amino acid long peptides examined by NMR. The fact that the phospho-status of PIN1 HL plays a role in the polar delivery of the auxin efflux carrier was well established. However, mechanistic understanding of the protein level is still largely lacking. Therefore, the published report of conformational changes in the loop that impact PIN sub-cellular routing re-fueled our curiosity and motivation. First, we asked if the PIN1 HL has any structure. 

We expressed and purified only a 25 kDa fragment of the hydrophilic loop of PIN1, representing about 50% of the entire loop (see Figure 1A and Supplementary Figure S1A), and designated it as HL–1 in this manuscript. To obtain the first structural information about PIN1 HL–1, we examined it with circular dichroism (CD), a fast method for determining a secondary structure of proteins [36]. 

The measured spectra of PIN1 HL–1 (Figure 1C) reached the first minimum at 229 nm, followed by negative maxima at 220 nm. Later, up to 200 nm, the signal was only decreasing. When compared with the control measurement of poly–L–lysine, which at pH seven is completely unstructured (Figure 1B, black line), the spectral curves of PIN1 HL–1 and control do not overlap, indicating that the HL is not completely unstructured. What is more, the PIN1 HL–1 spectrum corresponds neither to the spectra of poly–L–Lys in the α–helical nor in the β-sheet state (Figure 1B, red and blue lines, respectively), pointing out that PIN1 HL–1 cannot be defined as completely structured either. Unfortunately, the CD cannot provide precise structural data, only a gross composition with the share of alpha, beta, and unstructured regions without determining their exact locations. Therefore, we decided to zoom in on particular areas testing shorter fragments also outside the HL–1 to get more complete coverage of the whole PIN1 HL. 

For further detailed measurements, peptides 1 and 3 were chosen because computational modeling indicated them as primarily alpha-helical [37] (Supplementary Figure S1). Their largely negative CD spectra (Figure 1D, dashed lines) partially agreed with the modeling, unlike peptide 2, with a positive maximum around 225 nm (Figure 1D, solid black line), which resembles more the unstructured spectrum of poly–Lys (Figure 1B, black line) rather than the structured peptides. Notably, the computational prediction for peptide 2 included both alpha-helix and unstructured regions, and therefore its final spectrum could vary anywhere from pure α-helix to a completely unstructured protein. 

Considering that an alpha helix needs 3.6 amino acids per turn [38], another longer peptide, designated peptide 4, was selected from the PIN1 HL–1 sequence. Its structural prediction displayed five amino acids long helical region and two long unstructured flanks (Supplementary Figure S1). Like PIN1 HL–1, the spectrum of peptide 4 is characterized by a negative signal with local minima at 224 nm with a following local maximum at 219 nm (Figure 1E, dashed line), again indicating the presence of secondary structure elements.

Similarly, as for alpha-helix, β-sheet requires a minimum of 4 amino acids to form a plate [39]. Therefore, we subsequently also measured peptides longer than eight amino acids. Peptide 5 was also predicted to contain beta-sheet stretches (Supplementary Figure S1). Its measured spectrum indicates only a negative signal with local minima and maxima at 221 nm and 219 nm, respectively (Figure 1E, solid line). In summary, none of the peptides contained pure α-helix or β-sheet, as seen in the control spectra (Figure 1B). At the same time, except for peptide 2, which had the most significant share of disorder, all tested peptides could not be defined as entirely unstructured.

Regarding the fact that we did not find strictly defined structural elements while investigating longer and shorter fragments along the large loop of PIN1, we wanted to confirm or disprove the existence of secondary structures in the PIN1 HL. In globular proteins, α-helices or β-sheets undergo unfolding while heated; thus, their presence and gradual disappearance can be observed in a temperature gradient. Therefore, we examined the melting profile of PIN1 HL–1 using two different methods. Firstly, we analyzed the temperature effect on the secondary structure content in the far-UV CD spectra, which increased with the rise in temperature in which the protein was incubated (Figure 1F). This unexpected result suggested the presence and even greater abundance of secondary structures within the protein while being denatured. The changes were evenly distributed across the whole temperature range from 20 to 90 °C (for clarity, we show only curves for a few selected temperatures). We saw the same in the case of peptide 2 (Supplementary Figure S2C), which was the most disordered in previous measurements.

To clarify these not so easily explainable results, we changed the instrumentation and acquired a melting curve via nano Differential Scanning Fluorimetry (DSF). The nanoDSF registered three peaks at 59, 72, and 96 °C (Figure 1G, upper panel, black line, see arrowheads). The existence of the third peak at such a high temperature was especially unexpected. When one assumes a possible globular nature of the PIN1 HL, all unfolding events would typically happen at lower temperatures before reaching 96 °C. Surprising as it may be, still this result was consistent with the above-mentioned CD spectrum in temperature gradient (Figure 1F). Therefore, we wondered if we are not just seeing protein aggregation because of thermal denaturation and misinterpret it as the appearance of secondary protein structures. However, the precipitates of PIN1 HL–1 during thermal treatment were never observed, and the fragment was thermally quite stable (Supplementary Figure S2B). To verify the presence or absence of aggregates in a more analytical way, we investigated the nanoDSF scattering data that did not display any peak compared with the control measurement of buffer (Figure 1G, lower panel). This indicated that no significant aggregation occurred during melting. All those results suggest that we have secondary structures in the PIN1 HL, but they are not well defined and delimited. At least, we could not verify this with our peptides that only partially matched physical properties with their computational predictions. Thus, it is less likely that the PIN1 HL is a globular protein with a well-defined fold. On the contrary, the results indicate the properties of an intrinsically disordered (ID) protein. One of those is the increase in the strength of the hydrophobic interactions at high temperatures leading to stronger hydrophobic attraction driving the protein folding [40].

### 2.2. PIN1 HL Exists in a Monomeric and Dimeric State

The field of intrinsically disordered proteins is rapidly progressing, but the already published reports suggest that the ID regions in proteins can often serve as interaction platforms [41]. In connection with auxin efflux carriers, a recent study proposed that PINs may form disulfide-dependent dimers formed with the involvement of cysteines in the TMD regions. However, in this work, the authors did not exclude the participation of the hydrophilic loop in the process of dimerization [33]. For that reason, we tested if PINs hydrophilic loops could be involved in dimer formation. 

**Figure 2:**
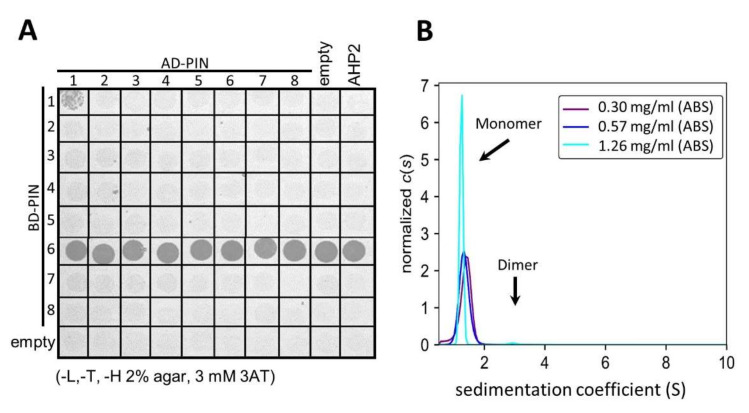
PIN1 hydrophilic loop homo-dimerizes while other long PIN loops do not. (**A**) A Yeast two-hybrid interaction assay of hydrophilic loops from PIN1 to PIN8 indicates the dimerization only for PIN1–HL. AHP2 as well as the empty plasmids containing only the activation domain (AD) and binding domain (BD) serve as negative interaction and growth control, respectively. Consistent auto-activation is observed for PIN6 HL. (**B**) Sedimentation velocity c(*s*) distribution analysis of PIN1 HL–1. The arrows point at peaks corresponding to the monomeric and dimeric state of PIN1 HL–1. The concentrations in the right corner are color-coded corresponding to the curves drawn in the c(*s*) distribution graphs.

First, based on membrane topology prediction, we cloned the complete HL loops of long PINs (PIN1–4 and PIN7), the intermediate length loop of PIN6, and the markedly shorter loops connecting the alpha-helices of endoplasmic reticulum (ER) PINs 5 and 8. Those constructs were then paired, by mating the yeast, in a matrix fashion, as depicted in Figure 2A. Thus, every PIN loop was paired with the other seven and itself. To exclude the auto-activation in the DNA binding domain (BD) containing constructs, we also paired them with an empty pDEST vector. As a second negative control, we used the *Arabidopsis thaliana* histidine-containing phosphotransfer protein 2 (AHP2), generally involved in cytokinin signaling. We did not expect any involvement of the PIN loops with the cytokinin phosphorelay [42] and congruently we did not detect any AHP2 vs. HL interaction. In the case of the PIN6 loop fused to the BD, we had consistent auto-activation of histidine synthetic genes visible as a row of spotted yeast colonies growing on the solid drop-out medium (see Figure 2A). In this assay we spotted cells on a medium depleted of leucine (−L), tryptophan (−T), and the crucial histidine (−H), which is only produced when an interaction between BD and activating domain (AD) is occurring. Although the PJ69–2A is one of the least prone to false-positive growth, to be even more stringent, we supplemented the medium with the 3–Amino–1,2,4–triazole (3AT) histidine synthesis inhibitor at 3 mM concentration. In this setup, we observed only PIN1 HL dimerization (top right corner of the yeast spot matrix). No other combination of PIN loops interacted in this assay (Figure 2A).

To verify this interaction with another non-yeast-based technique, we also utilized PIN1 HL–1 (see Figure 1) and tested it via analytical ultracentrifugation (AUC). As shown in Figure 2B, the sedimentation velocity experiment indicates the existence of major monomeric (~1.2 S) and minor dimeric (~3S) species. Unfortunately, the presence of dimers could be confirmed only at a higher concentration of PIN1 HL–1 (1.2 mg/mL). It cannot be excluded that such a high concentration of PIN1 HL is also present in the yeast nucleus during the Y2H assay. Notably, the HL–1 sequence was approximately 50% shorter than the entire PIN1 loop (see also Figure 1A) used for Y2H, as the expression levels of the longer loop in bacteria were too low for efficient purification. Therefore, the longer PIN1 HL may interact more strongly in yeast cells as it has additional regions that promote this interaction.

Since the analytical ultracentrifugation confirmed to some extent our results from Y2H, we wondered if the polar auxin transport inhibitor Naphthylphthalamic Acid (NPA) affects PIN dimerization. NPA has been recently reported to bind to the long *Arabidopsis* PINs 1–3 and 7, possibly at an interface of two crosslinking PIN moieties. It seems that the membrane-proximal conserved cysteine residues in the TMDs likely play a role in this PIN pairing, but the HL regions were not yet entirely excluded as NPA binding sites [33]. Therefore, we tested if NPA, at concentrations ranging from 10 to 50 µM, could affect PIN1 HL dimerization in the Y2H assay. To quantitatively evaluate yeast growth related to PIN dimerization strength, we used liquid culture in which optical density (absorbance at 600 nm) was measured. Unfortunately, the NPA did affect the yeast growth both in histidine- depleted (used for interaction testing) and histidine-containing (only −L, −T drop-out media; selection of cells containing both AD and BD encoding plasmids) media (Supplementary Figure S3A). To exclude the possibility of the NPA solvent causing yeast growth impairment, we tested liquid culture with various dimethylsulfoxide (DMSO) additions (Supplementary Figure S3C). We saw some effect on unmated PJ69 strain growth in rich YPD media when the DMSO content reached 1%. Still, in our experimental setup, DMSO concentration did not exceed 0.1% when the liquid culture was supplemented with NPA up to 100 µM. These results make it impossible to fully evaluate the effects of NPA on PIN1 HL dimerization. Instead, they hint at unspecific effects of NPA on yeast growth. We did not observe a striking growth decrease when yeasts were cultured in rich media supplemented up to 100 µM NPA (Supplementary Figure S3B), indicating an unspecific inhibition of yeast growth, mostly in the drop-out medium. Altogether, we could not conclude if NPA inhibits the HL–HL interaction in the Y2H assay as yeast growth, as such, is visibly affected by NPA. A non-yeast-based or maybe an in vitro assay will be needed in order to resolve this issue in the future.

### 2.3. PIN1 HL–GFP Overexpression Alters the Trafficking of Long PINs and Causes PIN1–Related Developmental Defects

Since our data indicated the possible function of PIN1 HL as a dimer interface, it seems likely that PIN1 HL is also an interaction platform for other cytoplasmic proteins. The PIN1 hydrophilic loop is natively in the cytoplasm [11] where it can also interact with coat proteins [16,43,44]. Considering the results above indicating the IDP nature of the PIN1 HL, its role as an interaction platform fits well [41,45]. Therefore, we decided to test possible interactions of HL with partners in the cytoplasm, hoping that overexpressing the loop might cause some problems in PIN trafficking or its localization at the PM.

**Figure 3:**
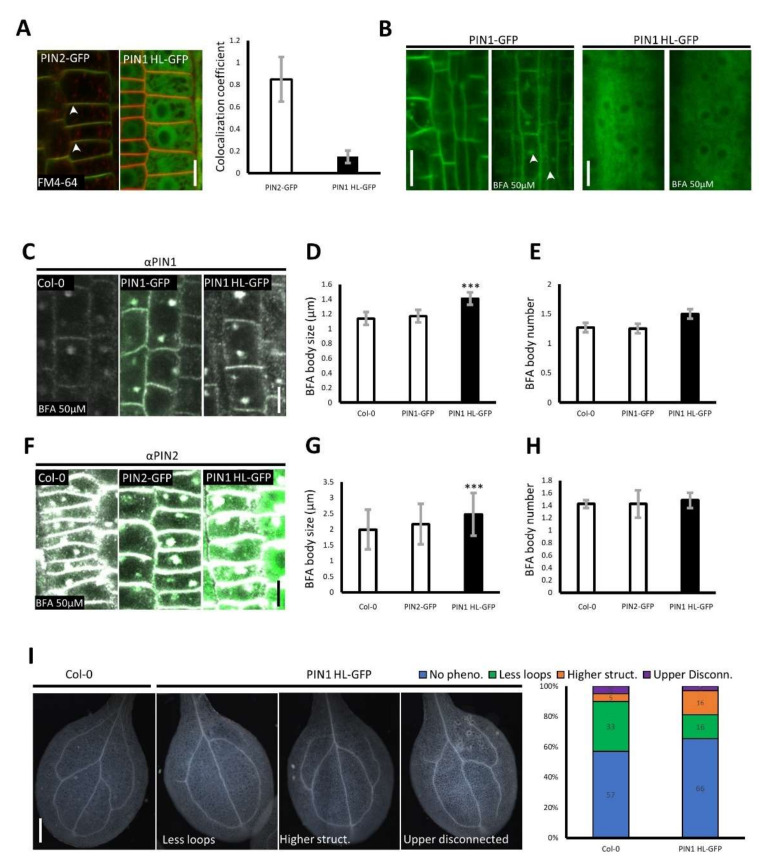
PIN1 HL–GFP overexpression alters the BFA-visualized PIN accumulation and causes defects in cotyledon vasculature development. (**A**) Representative confocal images of root epidermal cells expressing either PIN2–GFP or PIN1 HL–GFP and stained with FM4−64. Arrowheads indicate colocalization (yellow) of GFP (green) and FM4–64 (red) signal in PIN2–GFP control while no obvious colocalization is observed for PIN1 HL–GFP. The right panel represents colocalization coefficient quantification. The scale bar is equal to 5 µm. (**B**) Representative confocal images of root vasculature cells visualizing PIN1–GFP and PIN1 HL–GFP before and after BFA (50 µM). One-hour BFA treatment does not reveal the characteristic (arrowheads) BFA-induced aggregates in the PIN1 HL–GFP expressing line. Scale bars represent 10 µm. (**C**–**E**) Immunolocalization of PIN1 in root vasculature in Col–0, PIN1–GFP, and PIN1 HL–GFP after 1 h of 50 µM BFA treatment indicates significantly increased BFA body size in the PIN1 HL–GFP line. The right panel depicts the quantification of BFA body size (**D**) and number (**E**). Error bars represent standard deviation while the two-tailed *t*-test marks significance (*** *p* < 0.005). (**F**) Immunolocalization of PIN2 in epidermal cells in Col–0, PIN2–GFP, and PIN1 HL–GFP after 1–h and 50 µM BFA treatment indicates a bigger BFA body size in the PIN1 HL–GFP expressing line. GFP signals are represented in green while the immuno-stain signals are white. Quantification of BFA body size (**G**) and number (**H**) are depicted and evaluated statistically analogically as for PIN1 above (**D**,**E**). Scale bars for panels (**C**,**F**) equal 5 µm. (**I**) Representative images of cotyledon venation pattern in Col–0 and PIN1 HL–GFP seedlings. The right panel represents the quantification of venation changes in the PIN1 HL–GFP line compared to Col–0 (n < 80 for each line). Scored phenotypes: no phenotype, less loops, higher structures, upper disconnected. Scale bar: 200 µm.

Therefore, we generated an *Arabidopsis* line harboring the free hydrophilic loop of PIN1 fused C-terminally with GFP. The HL dimerized in the Y2H and AUC experiments. Consequently, we performed PIN1 HL colocalization with the membrane dye FM 4–64 to test if the loop would overlap with membranes that contain the native PIN1 or with other membranes, as the HL participates in events happening during vesicle budding, such as µ-adaptin binding. We registered a very low colocalization coefficient for PIN1 HL–GFP and FM4–64, in contrast to the clear PM overlap of the red styryl dye with the signal of PM integral PIN2–GFP used as a control (Figure 3A) [46]. Although these results indicate that PIN1 HL–GFP localizes mainly in the cytoplasm, there was still a possibility that it could interact with the intracellularly localized loops of PIN1 moieties that are in the membranes of the endosomes. Upon treatment with the fungal toxin Brefeldin A (BFA), those endosomes are more visible as they aggregate to form bigger structures, the so-called “BFA bodies” [47]. However, even after 1–h treatment with 50 µM BFA, sufficient to aggregate the PIN1–GFP control (Figure 3B, left-hand side), we did not visualize PIN1 HL–GFP accumulations (Figure 3B, right-hand side). We also performed an analogical treatment to ensure that this is not due to imaging difficulties registering a green BFA body in a plain of green HL–GFP in the cytoplasm. This time we immunolocalized the native PIN1 and PIN2 in the genetic background of PIN1 HL–GFP (Figure 3C–H), and for both PIN1 and 2, we observed an increase in the BFA body size, but the total amount of BFA bodies stayed unchanged (Figure 3D,E,G,H). This led us to conclude the existence of possible trafficking-related defects at the Trans-Golgi Network (TGN) level. Subsequently, we wanted to investigate if problems at the TGN would impact the PM localization of long loop PINs, such as PIN1 and PIN2. Those carriers have been shown to cluster at the PM, a phenomenon that presumably limits their diffusion, facilitating their asymmetric localization in root cells. However, we did not see obvious clustering defects for the immunolocalized PIN1 or PIN2 in the background of PIN1 HL–GFP (Supplementary Figure S4A–C), which showed clear groups of signal maxima preferentially on one cell side and therefore resulted in a lower signal count. In contrast, apolar aquaporin PIP2a–GFP, used as a control, expectedly showed more evenly distributed signal maxima around the entire PM, which in our quantification method resulted in more maxima counted (see Supplementary Figure S4A,B right-hand side). Although the PIN1 maxima at the PM of the PIN1 HL–GFP line were not obviously disturbed and were well discernible, the immuno-staining of PIN1 exhibited more background signal in the cytoplasm, presumably due to the presence of the overexpressed cytoplasmic loop (Supplementary Figure S4A,B). Recently, a study proposed the importance of clusters’ presence for PM polarity maintenance [31]. Moreover, in cortex cells, PIN2 polarity naturally switches from basal to apical, and this process is directly connected to protein trafficking [48]. Considering the fact that the phosphorylation status of the HL also plays a major role in proper polar targeting, we looked at the apical versus basal localization of PIN2. Although in the cortex, any targeting defects are easy to observe [49], we did not detect any issues in the PIN1 HL–GFP line (Supplementary Figure S4C).

Even though the observed trafficking alterations in the PIN1 HL–GFP line did not impact the sub-cellular localization of PIN1 or PIN2, it could influence the proper plant development. A convergence point of PIN1 PM polarity and plant ontogenesis is vasculature formation [50]. Therefore, we examined the vasculature patterns in the PIN1 HL–GFP expressing line. Compared to the wild type, we detected aberrations, such as less vascular loops, extra branches, or loop disconnections, sometimes with newly emerging but not closed vascular conduits as well as disconnected upper vasculature loops (Figure 3I). No prominent deformations, such as defects in the root length or alteration of the gravitropism, were observed at the seedling level (Supplementary Figure S5A,C), supporting our previous examinations. Moreover, we noticed a slight but not significant increased number of lateral roots (LR) in the PIN1 HL–GFP seedlings (Supplementary Figure S5B). The full-grown plant did not exhibit any *pin1*-related phenotypes on the main stem or other flower malformations (Supplementary Figure S5D,E), indicating that the native PIN1 may counterbalance any eventual defects caused by overexpression of PIN1 HL. In conclusion, the observed aberrations in the PIN1 HL–GFP line [5] seem to be more PIN1 than PIN2 related. This is exemplified by the *pin1*-connected vasculature development aberrations and the absence of *pin2* characteristic gravitropism defects [51]. 

## 3. Discussion

For many proteins, the three-dimensional structure and proper folding are essential for their correct function [52]. However, there is a group of proteins that require intrinsically disordered regions to do so [53]. With the current expansion of artificial intelligence (AI)-based prediction software like AlphaFold [54], it may look like the need for structural studies will decrease. Yet, no matter how close the models are to reality, they still have to be subsequently verified. On the other hand, most structure prediction programs fail to give satisfying results when it comes to IDPs. Only recently, OdinPred—NMR-database-based software was launched, providing the most up-to-date and accurate results [55]. It becomes apparent that well-defined structures, which we know from globular proteins, are the foundation, but at this cutting edge, the knowledge frontier will shift towards capturing the dynamics of proteins with IDPs, too. 

Our experimental data complement the in-silico predictions and confirm the presence of secondary structures within the PIN1 HL. However, the loop fragment we tested does not have the characteristic thermal melting profile of a globular protein. This is easier to explain when we assume that the PIN1 HL is intrinsically disordered and therefore has a so-called “turned out” response to heat when during temperature gradient its structure becomes stabilized rather than unfolded [40]. When we monitored the thermal melting spectrum of the PIN1 HL in the far-UV CD, we observed the heat-induced formation of the secondary structure. Moreover, further analysis of the temperature effects on structural properties of PIN1 HL revealed characteristic peaks for the thermal melting of the secondary structures. Notably, the presence of three peaks (Figure 1G) instead of one during differential scanning fluorimetry together with the absence of any aggregations led us to conclude that PIN1 HL undergoes heat-induced folding with subsequent unfolding, which is one of the characteristics of IDPs. Additionally, it is important to discuss two points. First, we could only express a part (50% approximately) of the PIN1 large loop that has been previously used for antibody production [56]. A similar approach was also selected for studying the structure of the C-terminal domain of caldesmon [57], where only a fragment of the protein was analyzed. Second, it was shown that the membrane and membrane-like environment could induce alpha-helical structures in the peptides [58]. Therefore, the fact that we are missing the rest of the protein, especially the trans-membrane region of PIN1, and the membrane proximity, could affect the final secondary structure composition and result in more unstructured areas in the HL–1. Considering all the above, our measurements with multiple techniques were quite consistent and overall encouraged the view of PIN1 HL as an IDP.

More support for the HL IDP nature comes from interaction studies. Intrinsically disordered proteins are often at binding interfaces since they can change their conformation from disordered to ordered upon binding to a partner [45]. The interactor can be either another protein or the IDP itself, in which case homo-multimers are formed, as shown in the N-terminal transactivation (TAD) domain of p53 protein. Natively, the TAD domain is an intrinsically disordered protein; however, after binding it forms a helical structure [59]. Moreover, recently it was proposed that long PINs undergo homo-dimerization, where the TMDs most likely serve as interaction interfaces. Nevertheless, the possibility of the hydrophilic loop participation in PIN–PIN interaction was not excluded [33]. 

In this study, we observed HL dimer formation using two independent techniques—the Y2H and AUC (Figure 2). We also tested if other PIN loops could exist as homomeric or as heteromeric dimers with PIN1. Despite using 3AT-containing media that inhibit unspecific yeast growth, we still observed positive interaction only for the PIN1 HL. The analytical centrifugation partially supported the Y2H results. Yet, it needs to be pointed out that the dimer peak was only observed at the highest of the tested concentrations (1.26 mg/mL) but compared to the one corresponding to PIN1 HL monomeric form, it was barely noticeable. It is possible that in the yeast nucleus, the PIN1 HL reaches such quantities, making the dimerization more visible than in the AUC. Dimer formation at higher concentrations would not be surprising when one acknowledges the IDP nature of PIN1 HL. The IDPs change from the dissolved, highly flexible state into a more structured cluster of multiple patterns when the concentration of monomers is higher than the protein’s dissociation constant. Then, it is common for IDPs to shift to the dimeric form, which also helps them in liquid-liquid phase separation (LLPS). The bioinformatical analysis of the PIN1 HL sequence revealed the presence of a prion-like domain (Supplementary Figure S1B), one of the most common sequences present in IDPs and highly involved in liquid-liquid phase separation [60,61]. For transmembrane proteins, such as PIN1, LLPS might be essential for pairing with the cytoplasmic interactors into the biomolecular condensate [62] like the µ-adaptins during PIN1 endocytosis [16,60,61]. Such clusters could create a dynamic biochemical environment that further helps with interactor pairing or signal transduction [62]. Moreover, in some animal transmembrane proteins, such as the linker for the activation of T cells (LAT), phosphorylation of tyrosine residues triggers LLPS, which results in protein activation [63]. Without any experimental proof, we cannot speculate whether PIN1 HL is involved in the phase separation or not. However, there is a potential for it to be so, as it also undergoes phosphorylation [18,23]. When concluding the HL−HL pairing discussion, it is worth mentioning that we could not conclusively show that NPA specifically interrupts PIN1 loop dimerization. In a quite recent publication, authors reported NPA binding at the dimerization interface of long PINs but did not exclude the hydrophilic loops as NPA binding sites. However, the motifs still considered as binding sites were restricted to approximately 100 amino acids shared among the HLs of PIN1, 2, 3, and PIN6. In this research, we wanted to use the opportunity of having PIN1 HLs showing dimerization as a setup to test if NPA prevents this loop-loop interaction as it does for full long PIN moieties [33]. Unfortunately, we could not conclusively show an NPA-specific inhibition of PIN1–HL dimerization in the Y2H system. Already the NPA alone decreased yeast growth. Therefore, if it diminished PIN1 HL dimerization, the effect was weak and could not be entirely separated from the unspecific effect of NPA on the yeast growth. Regarding those results, the Y2H setup is not able to verify the initially formulated question. Since other NPA-protein binding interfaces were reported much earlier, such as ABCB1 and TWD1 [64], the broader scope of NPA interactions and effects is not entirely unexpected and has to be considered in the design of future NPA-protein interaction experiments.

As hinted already, the PIN1 hydrophilic loop harbors several signals necessary for recruiting adaptor protein complexes during vesicle formation and motifs important for trafficking from the ER [16,65,66]. It is conceivable that these motifs and disordered regions interact with yet undiscovered protein players while the PIN protein is at the PM, forming complexes that promote structure formation in the initially unstructured regions of HL. If the process is cooperative, then subsequent binding of interactors can strengthen the integrity of the whole complex that is then not easily perturbed. That might explain why we did not see very prominent phenotypes when we overexpressed the PIN1 HL–GFP in the cytoplasm. 

One might expect the formation of a *pin*-like stem when the PIN1 function is strongly disrupted. But we never observed such strong phenotypes in the PIN1 HL–GFP overexpression line, in the Col–0 background, except for vasculature development defects in cotyledons of seedlings (Figure 3I), indicating that the native PIN1 function at the PM is not disturbed. It is worth noting that the venation patterning requires PIN1 activity as well as correct subcellular trafficking to facilitate the polarity switches during vein formation [67]. Mutants, such as the *vascular network defective* (*van4*) that encodes a guanine nucleotide exchange factor (GEF) for Rab GTPase, regulate vesicle transport and determine the specificity of membrane fusion. VAN4 protein localizes at the trans-Golgi network/early endosome (TGN/EE) and shows aberrant recycling of the auxin efflux carrier. Similarly, as for PIN1 HL−GFP, the PIN proteins aggregate in bigger BFA bodies in *van4* [68], suggesting defects of PIN sorting at the TGN level. The levels of PINs in *van4* were decreased, but the polarity was not greatly perturbed as in PINOID or PP2A mutants [27]. Similarly, we also did not observe striking PIN polarity defects or the basal vs. apical polarity switch, which can be most easily observed for PIN2 in root cortex cells closer to the elongation zone [49] (Supplementary Figure S4). These results indicate that the PIN1 HL−GFP overexpression rather perturbs PIN accumulation at the TGN, and for both PIN1 and PIN2, it probably overloads the sorting machinery in general. However, once the polarity is established, it is not disorganized by the freely diffusing HL of PIN1. Therefore, the BFA treatment may act as an amplifier visualizing even subtle trafficking defects that generally do not result in strong morphological defects. This is seen in the *bfa-visualized exocytic trafficking defective1* (*bex1*), in which PM localization of PIN1 and the development are hypersensitive to BFA. This ARF1A1C mutant has more BFA bodies but shows only moderately shorter and less branched stem when grown in the soil. It is likely due to the redundancy of polarity establishing mechanisms [30,31], indicating that once PIN localization and clusters, or even PIN dimers, are formed at the PM, they are not easily disorganized. The use of PIN1 HL, and the selective defects it inflicts, might suggest that the decision where to traffic a particular PIN falls at the level of TGN and involves the hydrophilic loops, but the polarity maintenance, when already at the PM, is instead attributed to the TMDs [32,33].

The dimerization observed only for PIN1 also provokes a discussion. Why do just PIN1 loops pair and not the others? Is it possible that the PIN1 HL is somehow different from the other long PINs? The PIN1 has the broadest expression in the plant tissues, being present both in the root and the shoot; maybe then its loop has to have functionalities not present in the other HLs of long PINs. The uniqueness of PIN1 has already been shown by the overexpression or silencing of the peptidyl-prolyl *cis*/*trans* isomerase Pin1At, that only affects the polarity index of PIN1 but not PIN2, 3, or 7 [27]. Since PIN1 has to maintain its PM polarity in much narrower cells in the root vasculature than, for example, PIN2 in the epidermis, it is conceivable that PIN1 HL facilitated dimerization additionally limits its diffusion, helping the auxin carrier to remain asymmetric. In addition, only the PIN1 exhibited a connection to the ABCB19 at the PM. In the *abcb19* mutant, PIN1 protein was less abundant in the detergent-resistant microdomains at the PM [69]. Those deliberations delineated distinctions between the long PINs and indicated that the loop could be the domain that diversifies them functionally. At this point, we can only speculate, and more focused studies need to be conducted, presumably involving NMR and taking into consideration the IDP nature of PIN1 HL.

## 4. Materials and Methods

### 4.1. Plant Material and Growth Conditions

All *A. thaliana* lines are in Columbia−0 background. The *PIN1::PIN1–GFP* [8], *PIN2::PIN2–GFP* [70], and *35S::PIP2–GFP* [71] have been published previously. The *PIN1 HL–GFP* line was generated by cloning the PIN1 hydrophilic loop (amino acid position 158–460) between SalI and XmaJI sites into the pEPA–35S–GFP pBluescript derived vector [72,73], and then in the binary vector pMLBART [74]. The seeds were placed on plates with Murashige and Skoog (MS+) medium with 1% sucrose, 0.5% phytagel, and stratified at 4 °C for 2 days, and then transferred to the cultivation room with the 16-h-light/8-h-dark-light cycle at 21 °C. Seedlings were grown from 3 to 8 days, depending on the assay.

### 4.2. In Silico Bioinformatical Analysis

Secondary structures were modeled using the online modeling software Phyre2 [37]. The amino acid sequence of PIN1 HL used for modeling starts at position 153 and ends at position 482 of the amino acid sequence PIN1. Prion-like domains in PIN1 HL (amino acid position 153–482) were predicted using PLAAC (Prion-Like Amino Acid Composition) [75] using a hidden-Markov model (HMM) algorithm. 

### 4.3. Protein Expression, Purification, and Peptide Synthesis

*E. coli* strain BL21 (DE3) was transformed with pET28a (Abcam, Cambridge, UK) vector encoding wild-type sequence of PIN1–HL, amino acids 289–451 with C-terminal 6xHis. Protein expression was induced with 0.5 mM isopropyl β–ᴅ–1–thiogalactopyranoside (IPTG) for 3h. Protein was purified using standard protocol for Ni-NTA raisins (Invitrogen, Waltham, MA, USA) followed by size-exclusion chromatography with Superdex 75 pg column (GE Healthcare), using elution buffer (50 mM NaP pH 7.4 (di-basic 37.7 mM, mono-basic 12.3 mM), 150 mM NaCl). Protein purity and concentration were estimated by SDS PAGE and Bradford assay, respectively. The peptides were synthesized by the company (ProteoGenix, Schiltigheim, France) with >95% purity. Lyophilized peptides were solubilized in Phosphate-buffered Saline (pH 7.4) to a final concentration of 1 mg/mL.

List of peptides:

Peptide 1: YSRRSQ, peptide 2: HTDFYSMM, peptide 3: PLETEAEIK, peptide 5: SDIMSLDGRQPLETEAEIKEDGKLHVTVRR, peptide 4: FSFGNKDDDSKVLATDGGNNISNKTTQ

### 4.4. Protein Stability Assay

A protein stability assay was performed as previously described [76]. Reaction mixtures were prepared with 0.8 mg/mL of purified PIN1–HL and 0.8 mg/mL of purified PIN1–HL with the addition of 50 mM L-Arg + L-Glu. The assay was held at 30 °C for 5 days. Untreated protein kept at 4 °C was used as a control. Samples were run on SDS PAGE and subsequently stained with Coomassie Brilliant Blue.

### 4.5. Circular Dichroism

CD spectroscopy was performed on the instrument Jasco J-815 measuring at wavelengths 260 nm to 200 nm; 1 mm cuvette was used. Total protein concentration was 0.7 mg/mL for PIN1 HL–1 and 0.4 mg/mL for the short and long peptides. Spectra were acquired through continuous scanning, where 4 spectra were averaged for each repetition. Data were analyzed in Microsoft Excel. Plotted spectra are averages of three independent repetitions.

### 4.6. Nano Differential Scanning Fluorimetry

The melting curve of PIN1–HL was obtained in triplicate on Prometheus NT.48 (NanoTemperTechnologies, GmbH, Munich, Germany). Fluorescence at 330 nm and 350 nm was detected from 20 °C to 100 °C with an increment of 1 °C per 1 min, and excitation power of 90%. Acquired data were analyzed in Microsoft Excel, where the first derivative of 350 nm/330 nm ratio of fluorescence was plotted as a function of temperature. Melting curve and scattering are the results of an average of two independent experiments. 

### 4.7. Analytical Ultracentrifugation

Sedimentation velocity experiments were carried out at 42,000 rpm at 20 °C on a Beckman Coulter ProteomeLab XL-I analytical ultracentrifuge and two-sector An60-Ti rotor with the optical pathway of 12 mm (Nanolytics Instruments, Potsdam, Germany) following standard protocol [77]. PIN1 HL−1 at various concentrations (0.3 mg/mL, 0.57 mg/mL and 1.26 mg/mL) was used. Buffer was used as a reference (50 mM NaP pH 7.4 (di-basic 37.7 mM, mono-basic 12.3 mM), 150 mM NaCl). SV data were collected using the absorbance at 280 nm in continuous mode. 200 scans at 5 min intervals were acquired. Time-corrected data were analyzed in SEDFIT 16.1c [78] in terms of a continuous c(*s*) distribution of sedimenting species.

### 4.8. Yeast Two-Hybrid

For the interaction of PIN Hydrophilic Loops, the Yeast Two-Hybrid methodology was used as published previously [79]. For the Yeast-2-Hybrid (Y2H) interaction we used the PJ69–2A strain and paired up the constructs by matting the haploid mating types A with Alpha. PIN HLs coding sequences, including stop codon, were cloned into Gateway vectors pDEST22/pDEST32, resulting in genetic fusions to the GAL4-activating domain (AD) and GAL4-binding domain (BD), respectively. Following sequences were used (numbers depicting start and stop amino acid): PIN1—<158–460>, PIN2—<167–484>, PIN3—<167–482>, PIN4—<168–457>, PIN7—<167–460>, PIN6—<167–411>, PIN5—<152–200>, PIN8—<157–218>. AHP control was published previously [80]. 

### 4.9. Whole-Mount In Situ Immunolocalization and Quantification of BFA Body Formation, PIN Clustering, and Polarity

PIN immunolocalizations in primary root were performed as described [10]. The anti-PIN1 and anti-PIN2 antibodies [81] were used at 1:600 dilution. The above-mentioned sera were re-raised in rabbits against amino acid epitopes 288–425 and 189–477, respectively as described by [19,56]. Indicated peptides were expressed from vector pDEST17 and purified as N-terminally 6xHis-tagged versions. The secondary sheep anti-rabbit antibody coupled to Cy3 (Sigma-Aldrich, St. Louis, MO, USA) was diluted at 1:600. Confocal microscopy was performed using a Zeiss LSM 780 Airy confocal microscope. BFA assay was performed using 50 µM treatment for 1 h. Quantification of clusters was counted as a number of signal maxima per cell. PIN apicalization was quantified as the ratio between apical versus basal PIN localization. The size of the BFA bodies was measured as their diameter using ImageJ [82]. 

### 4.10. FM4−64 Staining and Colocalization

5-day-old light-grown seedlings were stained with 2 μM FM4–64 dye (Invitrogen) in MS+ liquid medium on ice for 6 min. The seedlings were washed at room temperature in an MS+ liquid medium, mounted, and observed after 30 min. The pictures were acquired using the Zeiss 780 Airy Confocal microscope and colocalization analysis was performed in ZEN black software.

### 4.11. Leaf Vasculature Pattern

6-day-old light-grown seedlings were used for leaf venation analysis. Both cotyledons were cut and used for the analysis. Cotyledons were cleared in 4% HCl and 20% methanol for 15 min at 65 °C, followed by a 15 min incubation in 7% NaOH and 70% ethanol at room temperature. Next, cotyledons were rehydrated by successive incubations in 70%, 50%, 25%, and 10% ethanol for 10 min each at room temperature, followed by incubation in 25% glycerol and 5% ethanol for 15 min at room temperature. Finally, cotyledons were mounted in 50% glycerol and observed using a bright field and differential interference contrast (DIC) microscopy (Zeiss Axioscope.A1). Photographs were taken with a camera (Axiocam 506) at 10× magnification.

### 4.12. Primary Root Length

6-day-old light-grown seedlings were used to measure the root length. The plates were scanned, and the root length was measured using ImageJ [82].

### 4.13. Lateral Root Density 

8-day-old light-grown seedlings were used to analyze lateral root density as a number of lateral roots per primary root length. Seedlings were monitored using a stereomicroscope (Olympus SZX16) to count the number of lateral roots. The plates were scanned, and the root length was measured using ImageJ [82]. 

### 4.14. Primary Root Gravitropism 

Plates with 3-day-old light-grown seedlings were turned 90° compared to the original gravitropic vector. Plates were scanned 24 h after gravistimulation and the primary root gravitropic bending angle was measured using ImageJ [82].

## Data Availability

All data supporting the findings of this study are available within the paper and within its Supplementary Materials published online.

## Supplementary Materials

The following supporting information can be downloaded at: https://www.mdpi.com/article/10.3390/ijms23116352/s1.
