# Supplementary material for: The Hydrophilic Loop of Arabidopsis PIN1 Auxin Efflux Carrier Harbors Hallmarks of an Intrinsically Disordered Protein
Source: Int J Mol Sci. 2022 Jun 6;23(11):6352. doi: 10.3390/ijms23116352 (PMC9181416; doi:10.3390/ijms23116352)
Supplement: Supplementary file 1 [file ijms-23-06352-s001.zip › ijms_supplementary figures_VB.pdf]

A

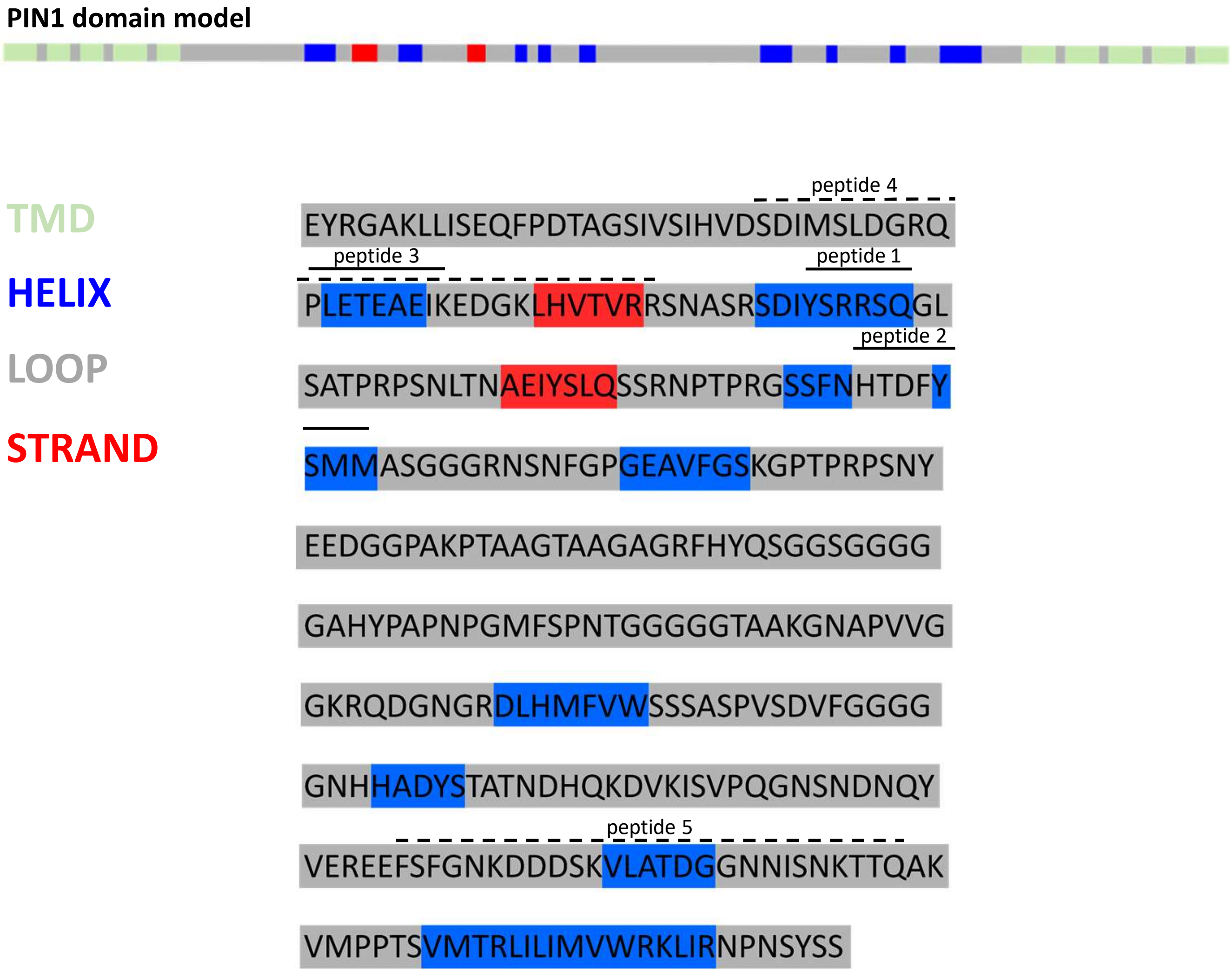

B

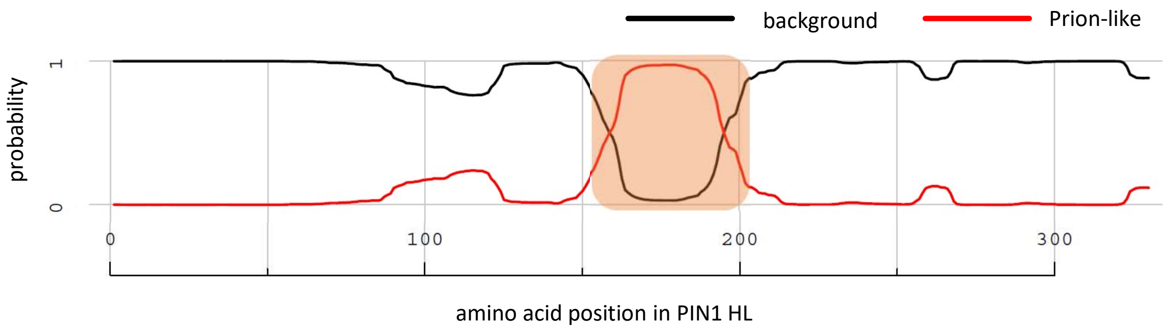

Figure S1: Bioinformatical analysis of PIN1 hydrophilic loop. **(A)** Computational modeling of PIN1 HL secondary structures. Alpha helix (helix, blue), beta-strand (strand, red), and unstructured regions (loop, grey) are highlighted within the amino acid sequence. The synthesized peptides (1-3) shorter and longer ones (4 and 5) are marked with solid and dashed lines respectively. The model of PIN1 with structural elements indicated and showing the predicted transmembrane domains (TMD) is visible on top for overview. **(B)** Identification of a probable prion-like domain within the PIN1 HL sequence using the PLAAC prediction program. Orange shading indicates a predicted prion-like sequence.

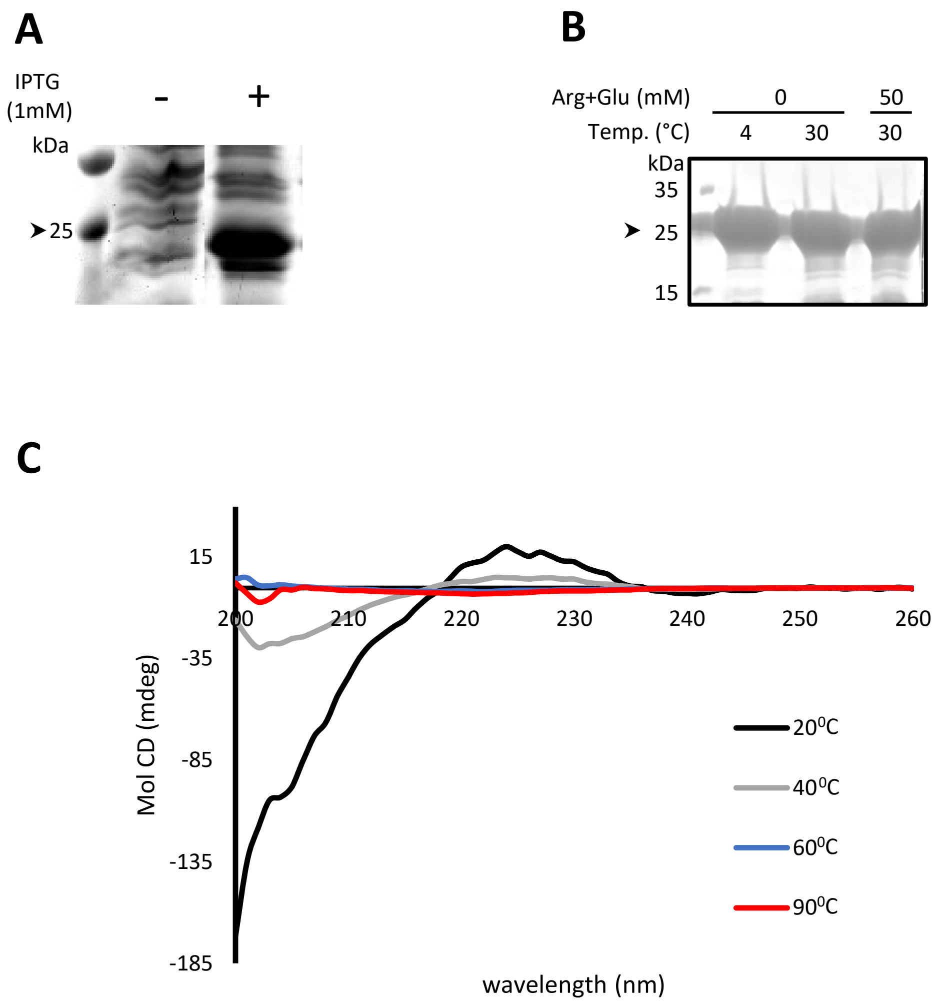

Figure S2: PIN1 HL does not degrade and retains some secondary structures even at high temperatures. **(A)** SDS polyacrylamide gel of recombinant PIN1 HL-1 expressed in *E. coli* BL21 (DE3) cells. Arrowhead indicates the PIN1 HL-1 band. **(B)** SDS polyacrylamide gel of an *in vitro* protein stability assay where purified PIN1 HL was incubated at 4 ^{0}C and 30 ^{0}C with or without the addition of the stabilizing agents (50mM L-Arginine and L-Glutamic acid). The intact band is indicating no major degradation even at higher temperatures. Arrowhead indicates the position of PIN1 HL-1 stained with the Coomassie Brilliant Blue. **(C)** Thermal melting CD spectra of peptide 2 obtained at different temperatures ranging from 20 to 90 ^{0}C indicate a decrease of random coil characteristics and increasing presence of secondary structures even at high temperatures which are features often exhibited by intrinsically disordered protein regions.

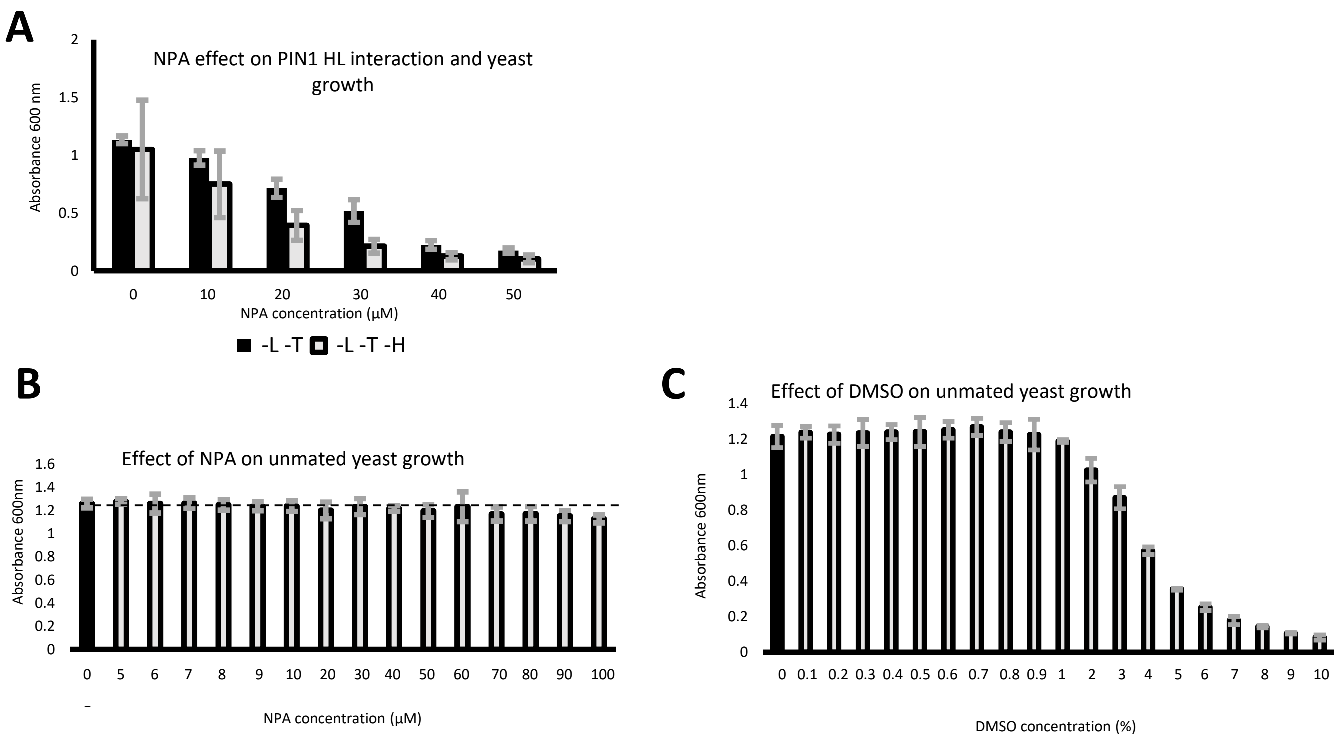

Figure S3: PIN1 HL dimerization in yeast is not discernably and specifically perturbed by NPA. **(A)** A yeast two-hybrid interaction assay in liquid media of PIN1 HL in presence of various NPA concentrations ranging from 0 to 50 µM, yeast growing only on leucine and tryptophan (-L, -T) drop-out media (black bars) are used as yeast growth control, the interaction is evaluated in media deficient also in histidine (-H; grey bars). NPA strongly affects the growth of yeast in both histidine-containing as well as drop-out media making the evaluation of its effects on PIN1 HL interaction impossible. **(B)** Effect of NPA, dissolved in DMSO, on unmated, untransformed yeast added to the liquid culture at various concentrations ranging from 5 to 100 µM. Yeasts growing without NPA addition were used as a control. **(C)** Effect of the common solvent DMSO alone at various concentrations ranging from 0.1 to 1% on unmated, untransformed yeast growth in liquid culture. Yeasts growing without the addition of DMSO were used as a control. Error bars in all graphs represent standard deviation. The PJ69 strain in (B) and (C) was grown in rich YPD media.

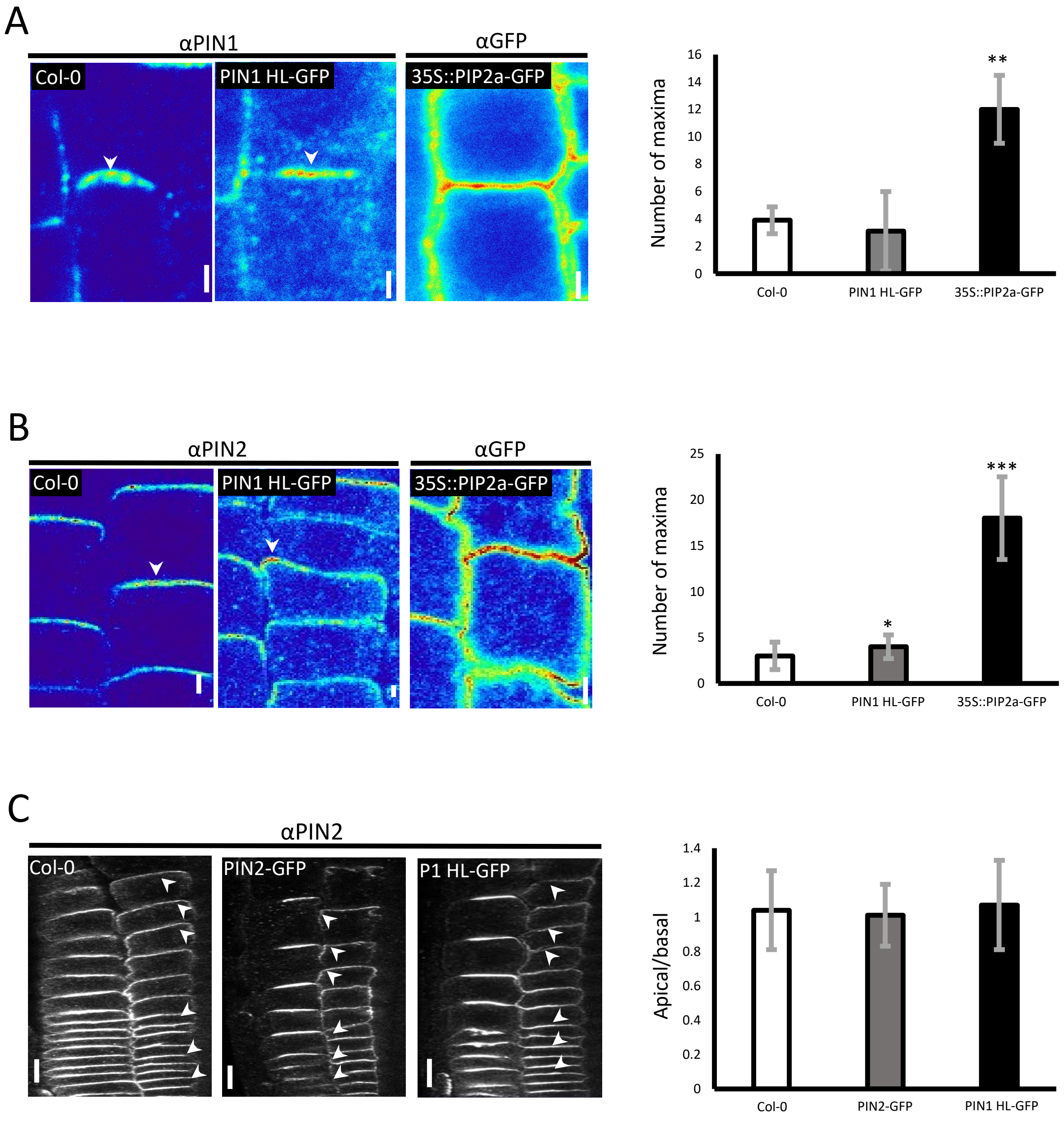

Figure S4: Overexpression of PIN1 HL does not strongly affect PIN clustering nor apical vs basal PM delivery. **(A)** Immunolocalization of PIN1 in Col-0 and PIN1 HL-GFP, and of GFP in 35S::PIP2a-GFP. The signal is color-coded. The right panel represents the quantification of signal maxima numbers per PM (cells depicted in A), more maxima correspond to less protein clustering on the PM. Error bars represent standard deviation. A two-tailed t-test marks significance ($p< 0.05$ **<0.005 ***). Scale bar: 2µm. **(B)** Immunolocalization of PIN2 in Col-0 and PIN1 HL-GFP, and of GFP in 35S::PIP2a-GFP in root epidermal cells. The signal is color-coded. The right panel represents the quantification of signal maxima numbers per PM (cells depicted in B), more maxima correspond to less protein clustering on the PM. Error bars represent standard deviation. A two-tailed t-test marks significance ($p< 0.05$ **<0.005 ***). Scale bar: 2 µm. **(C)** Immunolocalization of PIN2 in Col-0, PIN2-GFP, and PIN1 HL-GFP in root epidermal and cortex cells. Arrowheads indicate apical (shootward-pointing) or basal (root tip-pointing) polarity of PIN2. The right panel shows the quantification of the ratio between apical versus basal PIN2 localization. Error bars represent standard deviation. A two-tailed t-test marks the significance ($p<0.05$ **). Scale bar: 10 µm.

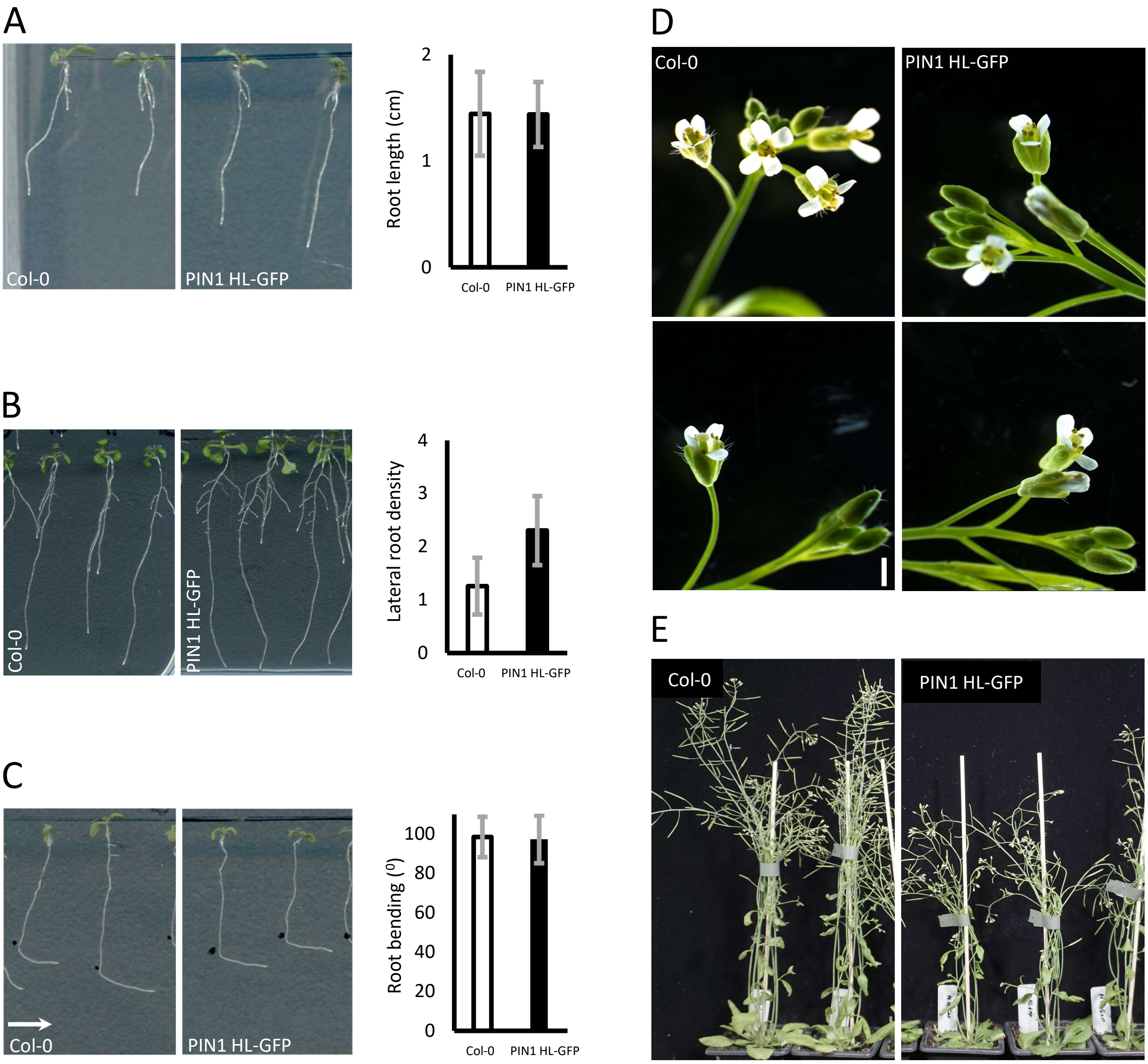

Figure S5: Overexpression of PIN1 HL does not cause strong pleiotropic developmental disruptions. **(A to C)** Root phenotyping of the Col-0 control and PIN1 HL-GFP. The right panel depicts representative quantification. Error bars represent standard deviation.**(A)** The primary root length of 6-day old seedlings is not significantly changed in the PIN1 HL-GFP line. **(B)** The lateral root density of 8-day old seedlings seems to be higher in the PIN1 HL-GFP line. **(C)** Primary root gravitropism of the PIN1 HL-GFP line does not show significant differences when compared to the Col-0 control (arrow represents the gravity vector). **(D)** The flower phenotype of the PIN1 HL-GFP line is devoid of visible defects. **(E)** Stems of 4-weeks old PIN1 HL-GFP overexpressing plants are shorter in comparison to Col-0.
